# Pulmonary artery sarcoma and severe valvular diseases in late-septuagenarian women: was 2-stage surgery an appropriate strategy? A case report

**DOI:** 10.1186/s40792-023-01805-6

**Published:** 2024-01-08

**Authors:** Sakiko Sato, Hideo Ichimura, Keisuke Kobayashi, Shuntaro Kawabata, Tomoyuki Kawamura, Hisashi Suzuki, Akito Imai, Kanji Matsuzaki, Akiko Sakata, Daisuke Matsubara, Yukio Sato

**Affiliations:** 1 Department of Thoracic Surgery, Hitachi General Hospital, Hitachi, Ibaraki 317-0077 Japan; 2 Department of Thoracic Surgery, Institute of Medicine, University of Tsukuba, 1-1-1 Tennodai, Tsukuba, Ibaraki 305-8575 Japan; 3 Department of Cardiovascular Surgery, Hitachi General Hospital, Hitachi, Ibaraki 317-0077 Japan; 4 Department of Pathology, Hitachi General Hospital, Hitachi, Ibaraki 317-0077 Japan; 5 Department of Diagnostic Pathology, University of Tsukuba, Tsukuba, Ibaraki 305-8575 Japan

**Keywords:** Pulmonary artery sarcoma, Valvular disease, Two-stage surgery, Surgical strategy, Surgical approach

## Abstract

**Background:**

Pulmonary artery sarcomas (PASs) are rare, and complete tumor resection is often difficult at the time of detection. We encountered a case of PAS that was thought to be resectable; however, the patient had severe symptomatic valvular disease. We faced a difficult decision regarding the surgical strategy.

**Case presentation:**

A 76-year-old female presented with a history of polysurgery for multiple primary cancers. She was referred to our department with a calcified mass in the right pulmonary artery (PA) and severe symptomatic valvular disease. After a discussion with the cardiovascular surgeon, we decided to perform a two-stage surgery. She underwent valvuloplasty through a median sternotomy, resulting in an improvement in her exertional dyspnea. The tumor was removed three months later with a right upper lobectomy and PA patch reconstruction through a posterolateral thoracotomy. When the PA was opened, the edge of the tumor was entrapped by vascular clamp forceps because of insufficient dissection of the adhesions between the superior vena cava and the right main PA resulting from the first operation. The patient underwent proton therapy twice for chest wall metastases which recurred three months after surgery, and local recurrence in the PA was diagnosed five months after surgery. The patient was alive with stable disease 25 months after surgery.

**Conclusion:**

Two-stage surgery for PAS and valvular disease resulted in incomplete resection of the PAS in the right PA. It is important not to underestimate surgical adhesions due to the initial surgery and to consider and implement measures to prevent adhesions of critical vessels during the second operation.

**Supplementary Information:**

The online version contains supplementary material available at 10.1186/s40792-023-01805-6.

## Background

Pulmonary artery sarcoma (PAS) is rare. Although the prognosis of PAS is poor [1, 2], long-term survival has been reported in patients who underwent complete surgical resection [3] or in cases of a specific histological type [4]. We encountered a case of exertional dyspnea in which a calcified mass was detected in the right pulmonary artery (PA) that was suspected to be PAS and severe valvular disease. We discussed the surgical strategy with cardiovascular surgeons and decided on a 2-staged surgery preceded by valvular surgery.

## Case presentation

**Fig. 1:**
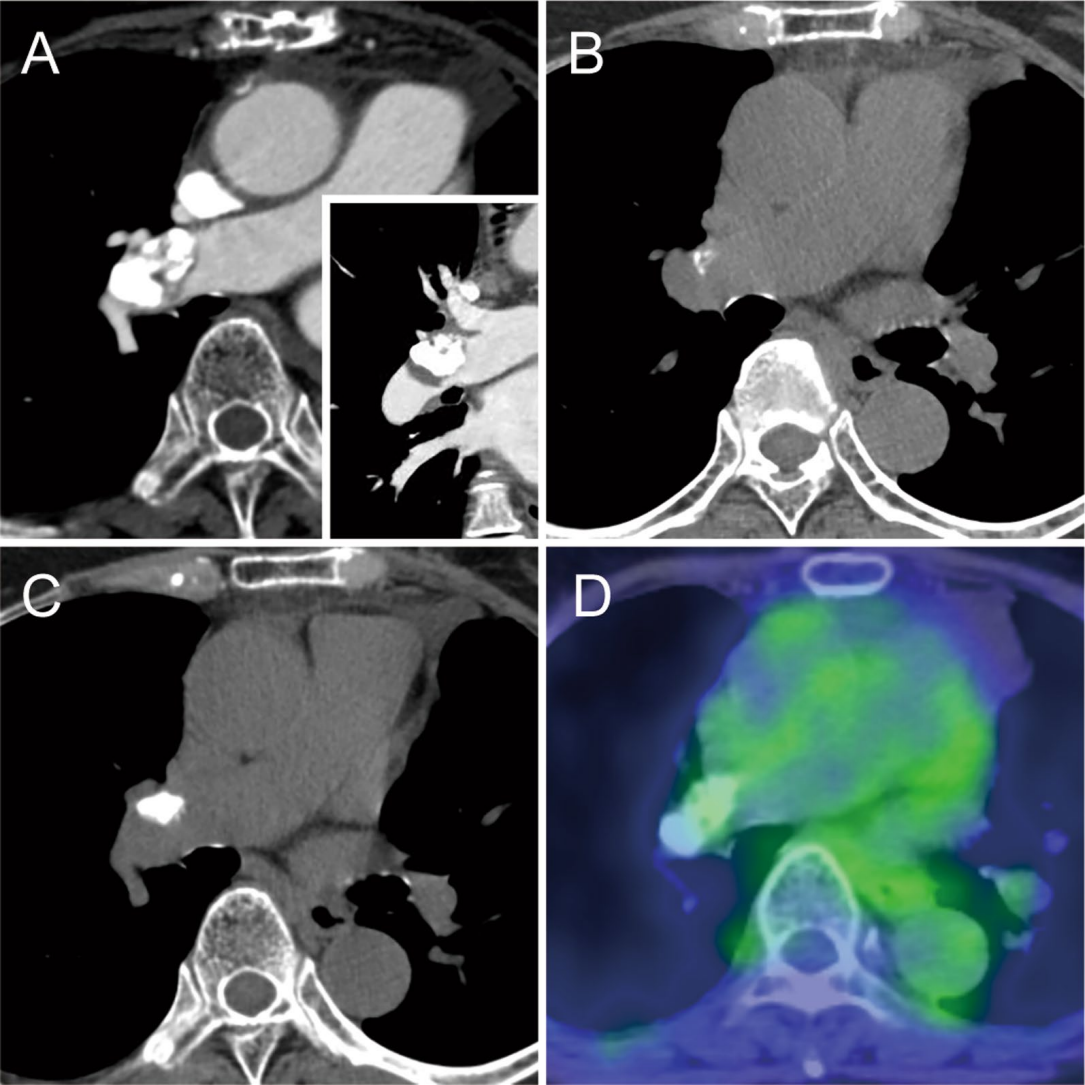
**A** Enhanced computed tomography (CT) showing a calcified nodule in the right pulmonary artery. Inset showing reconstructed frontal section. **B** CT performed 2 years before the first visit to our department showing a smaller calcified lesion. **C** CT performed 1 year before the first visit to our department showing an enlargement of lesion that was not recognized at this time. **D** The fluorodeoxyglucose (FDG) positron emission tomography CT showing abnormal accumulation of FDG in the lesion with a standardized uptake value max of 2.16

A 76-year-old female patient with exertional dyspnea was referred to our department with an enlarged calcified mass in the right PA. She had a history of multiple primary cancers and had undergone several surgeries: mastectomy for metachronous bilateral breast cancer (right at age 49, left at age 66), pulmonary resection (right S9 wedge resection and left S3a segmentectomy at age 56) for simultaneous bilateral lung cancer, and left nephrectomy for left renal cancer at age 65. She also underwent surgery for melanoma of the right finger at the age of 66. Chest computed tomography (CT) performed as a periodic examination 10 years after the surgery for renal cancer revealed a calcified nodule in the right PA (Fig. 1A). The calcified nodule showed slow enlargement over the previous two years, retrospectively (Fig. 1B and C) and was 25 mm in diameter, located just peripheral of the superior trunk of the right PA and the beginning of the A6 branch. Fluorodeoxyglucose positron emission tomography/CT (FDG PET/CT) showed that the calcified area of the nodule did not take up FDG, but the area of soft tissue density on CT showed uptake with a maximum standardized uptake value max of 2.16 (Fig. 1D). Preoperative echocardiography revealed severe mitral valve regurgitation (MR) with P2 prolapse and moderate tricuspid valve regurgitation (TR) with an ejection fraction of 81%. Regarding laboratory data, the creatinine level was 1.22 mg/dl, and tumor marker levels were unremarkable (Carcinoembryonic Antigen: 2.0 ng/ml, cytokeratin 19 fragment: 2.9 ng/ml, pro-gastrin-releasing peptide: 72.4 pg/ml).

We planned a 2-staged surgery preceded by valvular surgery under total extracorporeal circulation. The cardiovascular surgeons performed mitral and tricuspid valvuloplasty through a median sternotomy (operative time, 422 min; extracorporeal circulation time, 257 min). During the initial surgery, we encircled the superior vena cava (SVC) at the most central site in the pericardial sac. We opened the right mediastinal pleura and confirmed the presence of the nodule in the right PA by palpation and judged that it was difficult to reconstruct the PA, especially on the peripheral side of the PA, through a median sternotomy. The postoperative course was uneventful. Her exertional dyspnea improved three months after surgery, and the echocardiography showed no regurgitation.

**Fig. 2:**
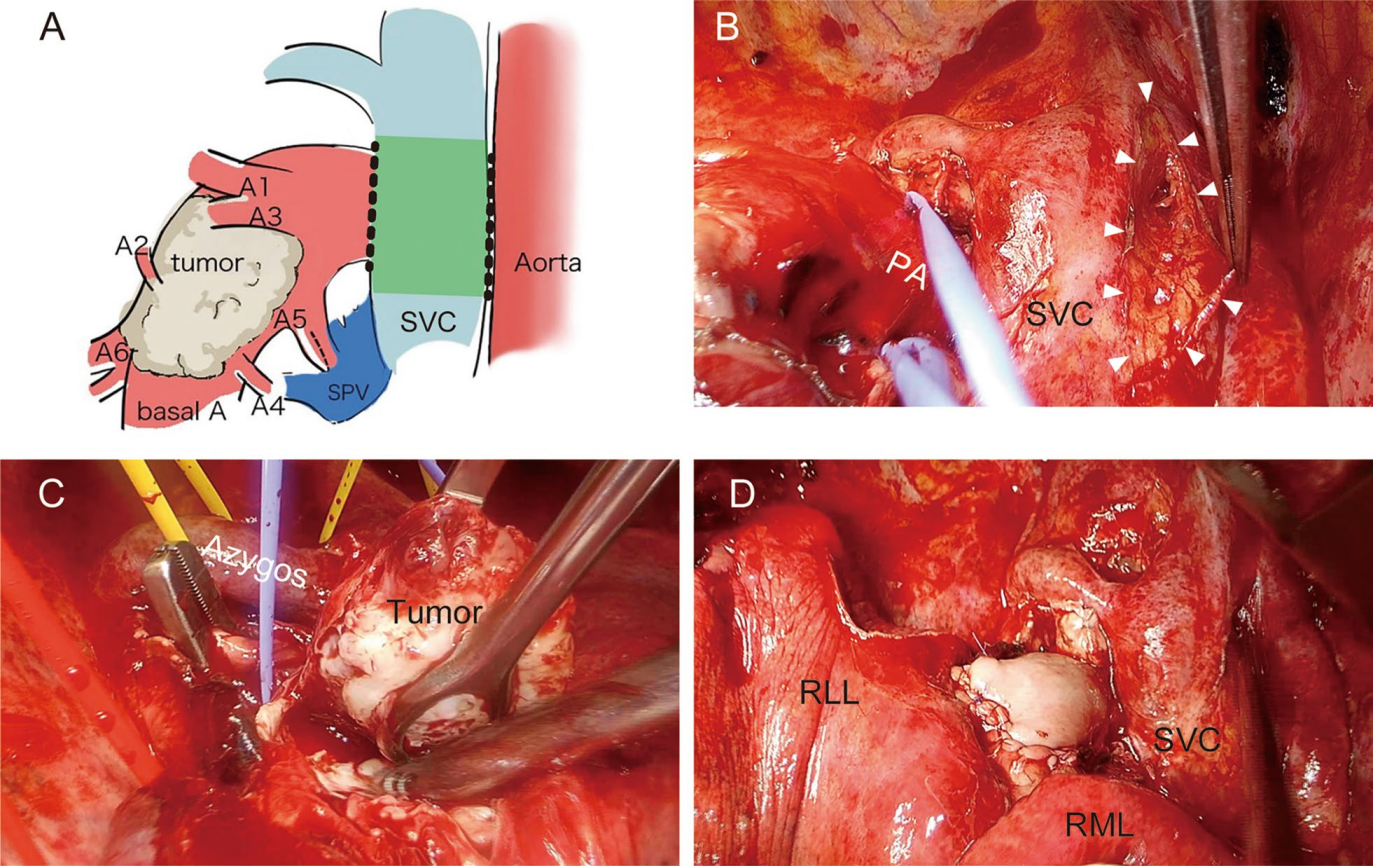
**A** A schema of intraoperative findings. The bold dotted line indicates severe adhesions. The greenish area of the SVC indicates areas where wrapping to prevent adhesion is anticipated in the Discussion section. SVC; superior vena cava, SPV; superior pulmonary vein. **B** Intraoperative image showing dense adhesive change in the area between the SVC and the aorta (arrowheads). PA; pulmonary artery, SVC; superior vena cava. **C** Intraoperative image showing the tumor in the pulmonary artery grasped with the forceps. **D** Intraoperative image showing the PA reconstructed with bovine pericardium. *RLL* right lower lobe, *RML* right middle lobe

Therefore, a second surgery was performed. Following posterolateral thoracotomy at the fifth intercostal space, a right upper lobectomy was performed. The tumor was identified as a stony hard mass in the right PA. To clamp the right main PA, we tried to dissect apart the SVC and the right main PA and also tried to approach the right main PA for encircling from the space between the SVC and the aorta. However, severe adhesions obstructed dissection. Although the surgical margins appeared to be insufficient, we were able to grasp the mass manually. Therefore, we decided to place a vascular clamp forceps on the center of the mass, as centrally as possible. After introducing heparin with an activating clotting time of 200–250 s, the right main PA, middle pulmonary vein, and inferior pulmonary vein were clamped. When we opened the middle PA trunk using Metzenbaum scissors, a white stony mass that did not adhere to the artery wall on the mediastinal side was removed with the adherent PA wall. On the central side of the mass, the soft tissue contiguous to the mass was trapped by vascular clamp forceps. Because we could not loosen the forceps, the pinched tissue was separated, and the tumor was removed. The defect in the right main PA was reconstructed with bovine pericardium (Fig. 2) (Additional file 1: Video S1). The operative time was 404 min, with a blood loss of 940 ml. The postoperative course was uneventful, and the patient was discharged on postoperative day 16.

**Fig. 3:**
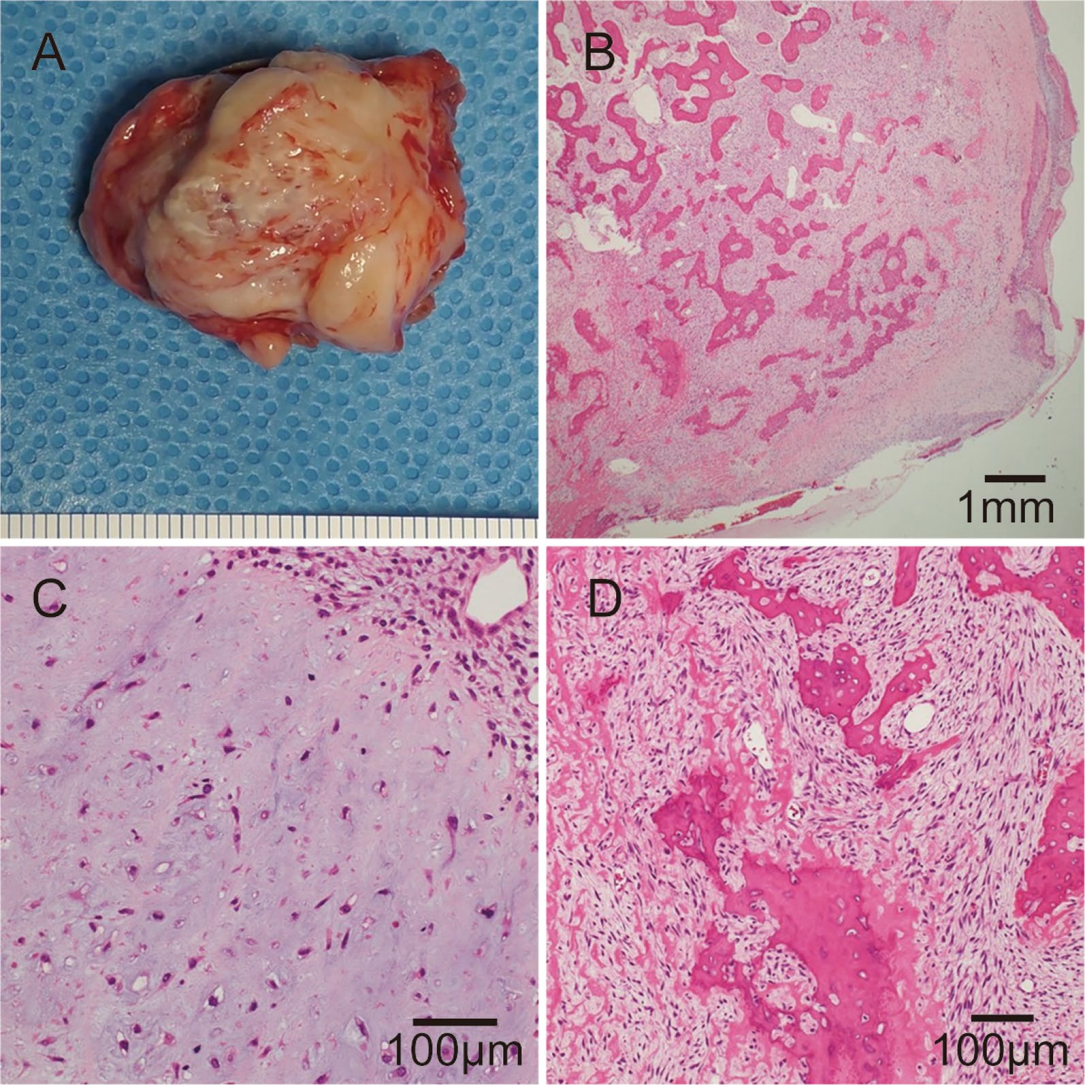
**A** Photograph of the removed specimen. Histopathological microscopic image showing osteosarcoma component (**B**), chondrocyte component (**C**), and spindle cell component (**D**) of the tumor

Histopathological examination of the tumor revealed that the pulmonary artery intimal sarcoma consisted of three components: osteosarcoma components, chondrocyte components, and spindle cell components (Fig. 3A–D).

**Fig. 4:**
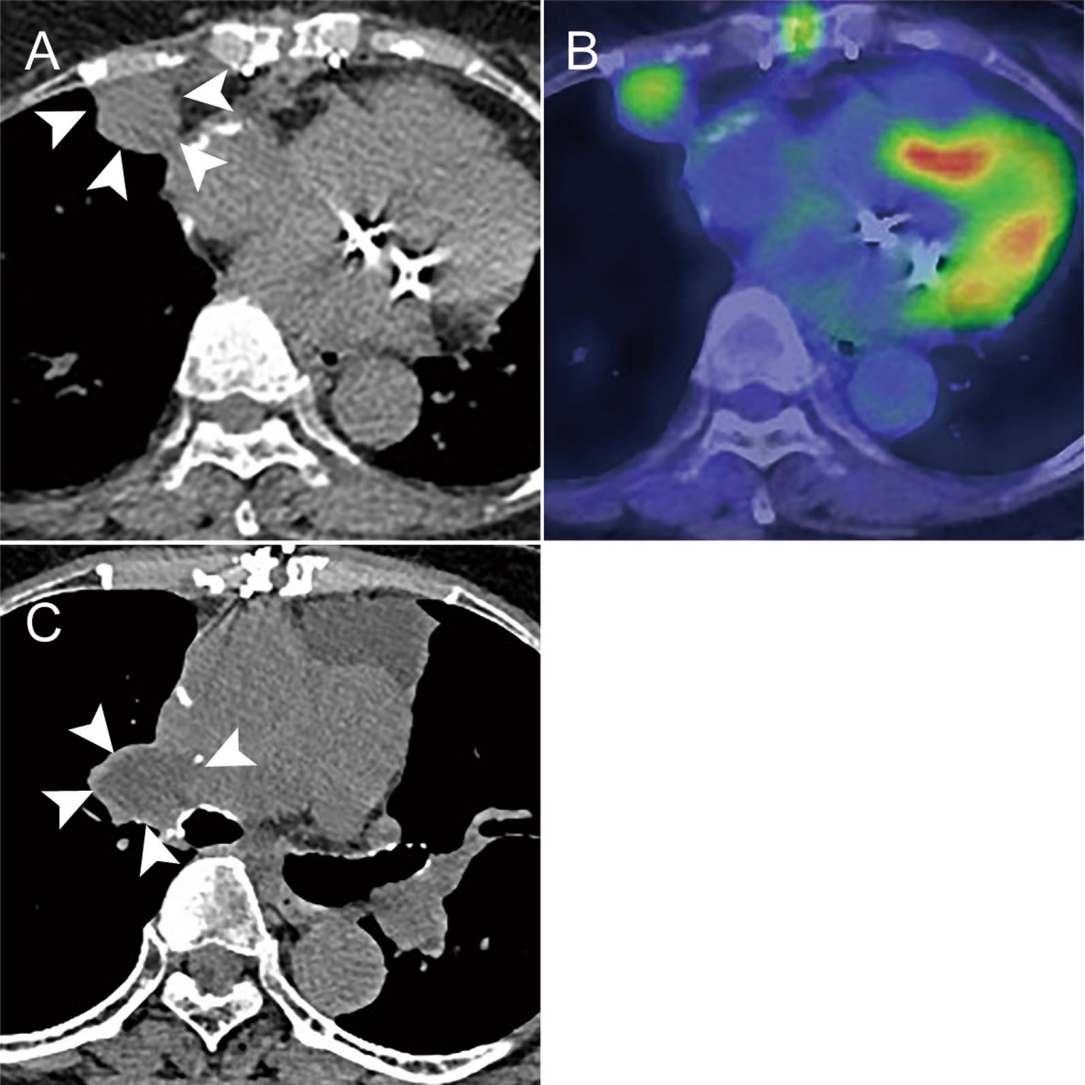
**A** Computed tomography (CT) obtained three months after the tumor removal showing a nodular lesion adjacent to the chest wall (arrowheads). **B** The fluorodeoxyglucose (FDG) positron emission tomography CT showing abnormal accumulation of FDG in the lesion. **C** CT obtained five months after the tumor removal showing a low-density lesion in the right pulmonary artery (arrowheads)

Three months after the second operation, FDG PET/CT revealed a hypodense lesion without calcification in the right chest wall (Fig. 4A). CT-guided biopsy revealed PAS recurrence. FDG PET revealed another slight accumulation in the right PA in the central region of the PA reconstruction. Genomic analysis using the FoundationOne^®^ companion diagnostic revealed a low-tumor mutational burden (TMB), microsatellite stable, and the loss of the methylthioadenosine phosphorylase (MTAP) gene (MTAP loss). According to a multidisciplinary conference, proton therapy was administered only to the chest wall lesion, and short-interval observation was determined for the lesion of the right PA because it was difficult to distinguish reactive accumulation associated with PA reconstruction from local recurrence. Two months after proton therapy, CT revealed a low-density lesion in the PA, indicating local recurrence (Fig. 4B). She received a second proton therapy for local recurrence and is alive independently with stable disease 25 months after tumor removal. At home, she maintains almost the same level of daily life activities as before the two surgeries with doing housework.

## Discussion

We report a patient with PAS in the right main PA and symptomatic severe valvular disease who underwent 2-stage surgery preceded by valvular surgery followed by tumor resection. This strategy resulted in incomplete PAS resection. A surgical case series reported that [1–3] most patients were aged 30–60 years and had lesions that included the main PA. The most commonly performed procedure is pneumonectomy or pulmonary endarterectomy with cardiopulmonary bypass through a median sternotomy. The present case was atypical and rare in terms of age, tumor location, extent, and comorbidities.

The reasons for our decision to perform a two-stage surgery were as follows. First, the patient’s preference was to improve shortness of breath on exertion, and she was reluctant to have the tumor in the PA removed. Second, based on the clinical course of the symptoms, the shortness of breath was more likely attributable to the valvular disease than to the tumor in the right PA. Third, the patient was a late septuagenarian, and since we assumed the possibility that bi-lobectomy (right upper and right middle lobe) might be necessary depending on the surgical findings, we considered that a one-stage surgery including double valvular surgery, bi-lobectomy and tumor removal with PA reconstruction would be too invasive and difficult to tolerate and could result in a major decline in quality of life. Additionally, we were not sure of the optimal approach for the one-stage procedure. Even with median sternotomy plus a lateral incision, we concluded that it would be difficult to reconstruct the PA, especially on the peripheral side. If her dyspnea on exertion did not improve after the first valvular surgery, we planned to discuss the treatment options with the patient again. However, after the initial surgery, her symptoms improved, and we decided to proceed with tumor removal.

The main reason for incomplete resection was the impossibility of dissection between the SVC and the right main PA. When we planned the 2-stage surgery, we anticipated that we would be able to dissect between them, even after surgery, using total extracorporeal circulation. Moreover, because the central side of the lesion could be held in hand intraoperatively, we determined that the PA could somehow be clamped on the central side of the tumor, which turned out to be incorrect, as revealed intraoperatively.

Regarding the optimal strategy, the clamshell approach may have allowed for one-stage valvuloplasty, removal of the PA tumor, and PA reconstruction [5]. If this approach is performed under complete cardiopulmonary bypass, it would be advantageous for the surgeon to open the PA and directly evaluate the presence of luminal skip lesions for complete resection. Alternatively, complete resection would have been possible if we had initially performed tumor removal via a posterolateral thoracotomy without valvular surgery. However, this was an option that could not be decided on. Regarding the posterolateral thoracotomy approach, we recognized the advantage of suturing, especially on the dorsal and peripheral side of the PA patch plasty, based on our experience in this case. In addition, we discussed the utility of the MitraClip^®^ percutaneous therapy as a less invasive approach [6]. Since the present case had a moderate TR requiring tricuspid valvuloplasty, MitraClip^®^ therapy was not considered to be an appropriate option. Furthermore, the need for anticoagulant therapy after MitraClip^®^ therapy would be a concern for the following tumor removal. It is important not to underestimate surgical adhesions due to the initial surgery and to consider and implement measures to prevent adhesions between critical vessels during the second operation. For example, because a polytetrafluoroethylene (PTFE) sheet has been reported to be useful as an adhesion barrier [7], wrapping the SVC with a PTFE sheet during the initial surgery in the area illustrated in Fig. 2A is easy to perform and might be effective in preventing adhesion between the SVC and right PA.

In terms of the treatment of recurrent lesions, we discussed the patient with multidisciplinary molecular tumor boards using a comprehensive cancer genomic profiling test. The tumor cells did not show any indications for immunotherapy, such as high TMB or microsatellite instability [8]. Although the tumor cells showed only MTAP loss, we concluded that there were no druggable mutations. There are some case reports showing the effectiveness of cytotoxic anticancer agents (amrubicin and ifosfamide, amurubicin and carboplatin, and ifosfamide and epirubicin) in patients with pulmonary artery sarcoma aged 40–60 years [9–11]. However, we concerned about cardiac toxicity of anthracycline drugs and the fact that this case was older than previously reported cases. After discussing the post-proton therapy medical plan (anticancer therapy with amrubicin monotherapy or palliative follow-up) with the patient and her family, she preferred palliative follow-up, is living independently at home, and visits our outpatient clinic periodically.

## Conclusion

Although we remain uncertain about the optimal strategy in this case, we report it in the hope that it would enhance the preoperative discussion to achieve complete resection for surgeons who will face a similar situation in the future.

### Supplementary Information

**Additional file 1: Video S1.** Intraoperative video showing tumor removal from the right pulmonary artery and patch reconstruction of the bovine pericardium

## Data Availability

All data generated or analyzed during this study are included in this published article.

## Abbreviations

CT: Computed tomography
FDG: Fluorodeoxyglucose
FDG PET/CT: Fluorodeoxyglucose positron emission tomography/computed tomography
MR: Mitral valve regurgitation
PA: Pulmonary artery
PAS: Pulmonary artery sarcoma
SVC: Superior vena cava
TR: Tricuspid valve regurgitation
